# 

**Published:** 1916-02-12

**Authors:** 


### CONTENTS.

PAGE

**The Florence Nightingale Memorial** ... ... 421
**Professional Handwriting** ... ... ... ... 422
**Hospital and Institutional News** ... ... .. 423
The Florence Nightingale Memorial—The late Mr. Walter G. Carnt—The Royal Red Cross—Soldiers and Civil Hospitals—Where the Shoe Pinches—The Drama and St. Thomas's Hospital—An Opponent of the Asylum Atmosphere—A Birmingham Hospital Unit for the Front—The Order of St. John—The Last of the Indian Hospital—A Model for Hospital Speeches—Former Secretaries on Hospital Boards—The *Southern Cross* Again—Consulting Surgeons for Leeds Workhouse Infirmary—The Notification of Pregnancy—A Patient's Reception at Liscard Hospital—The Coroner's Irony—Another Red Cross Sale—The English in Italy—Trench Foot at the Front—The Royal Salop Infirmary—The Supply of Petrol for Doctors' Cars—The Shortage of Doctors—Public Pharmacists' New Chairman—Drug Contract for a Military Hospital—This Week's Drug Market—Will Every Reader Please Help?
**A Medical Causerie** ... ... ... ... ... 429
**Legal Notes for Institutional Workers** ... 430
**Some Military Medical Economies** ... ... 431
The Value of Criticism.

PAGE

**Domestic Economy in the Isolation Hospital** 432
An Example of Prudent Housekeeping.
**The Department of Nursing** ... ... ... 433
Matrons and Sisters.
**King's College Hospital** ... ... ... ... 435
Some Nursing Developments.
**Recent Attacks on the Uniform System** ... 437
Some Criticisms and Suggestions.
**Leeds General Infirmary** ... ... ... ... 438
**Reports on Hospitals of the United Kingdom** 439
By Sir HENRY BURDETT, K.C.B., K.C.V.O.
The New Hospital Wards at Newcastle Union Workhouse.
**The Editor's Letter-Box** ... ... ... ... 440
**Passing Events and Topics** ... ... ... ... 440
**Royal Infirmary, Edinburgh** ... ... ... 440
**Leicester's Magnificent Red Cross Effort** ... 440
**The Institutional Worker** ... ... (Suppl.) 1
How to Utilise the Lady Helper: Answer to January Question.
Questions for February: (1) A Hospital or Institution Invention—(2) Economies in the Matron's Department.
Questions Invited.
Enquiries and Answers.
Employment and Training.
The Housekeeper's Department.
Miscellaneous.

